# Acetic Acid and Iodine Staining for Determining Malignancy in Solid Tumors

**DOI:** 10.31557/APJCP.2021.22.2.463

**Published:** 2021-02

**Authors:** Maulina Indah Anugrah Putri, Sonar Soni Panigoro, Agnes Stephanie Harahap, Trevino Aristarkus Pakasi, Bayu Brahma

**Affiliations:** 1 *Surgical Oncology Division, Department of Surgery, Faculty of Medicine, Universitas Indonesia, Cipto Mangunkusumo Hospital, Jakarta, Indonesia.*; 2 *Department of Anatomical Pathology, Faculty of Medicine, Universitas Indonesia, Cipto Mangunkusumo Hospital, Jakarta, Indonesia. *; 3 *Department of Community Medicine, Faculty of Medicine, Universitas Indonesia, Jakarta, Indonesia. *; 4 *Department of Surgical Oncology, Dharmais Cancer Hospital, National Cancer Center, Jakarta, Indonesia.*

**Keywords:** Acetic acid, breast cancer, frozen section, iodine, oral cancer, solid tumor, thyroid cancer, vital staining

## Abstract

**Objective:**

Surgical margin is an important prognostic factor in solid cancer surgery. Frozen section (FS), the gold standard for intraoperative surgical margin evaluation, requires extensive waiting time and expensive FS devices. The purpose of this diagnostic study was to verify whether multi-staining (MS) method with acetic acid and iodine could be used to differentiate malignant and non-malignant lesions of solid tumor.

**Methods:**

The study was conducted on patients with solid tumor who underwent surgery in the Surgical Oncology Division of Dr. Cipto Mangunkusumo General Hospital from December 2017 to April 2018. Samples measuring less than 5 mm, necrotic tissue sample, and patients who did not agree to participate in the study were excluded. Every specimen was divided into two, one side as unstained control and the other side as MS samples. MS samples were sprayed with 10% acetic acid combined with iodine. MS samples and unstained controls were sent for histopathologic results and the pathologist was blinded to group assignment. Acetowhitening reaction in the sample was an indication of a positive MS result, and the presence of malignant foci in the histopathology examination was classified as positive pathological results.

**Results:**

Five-hundred-and-twenty samples were obtained from 150 patients. MS method was found to have sensitivity and specificity of 82%, and 63.5%, respectively. In subgroup analysis, we found that MS method has a sensitivity and specificity of 100% and 79.3%, respectively for epithelial breast tumor; 65.7% and 83.3%, respectively for thyroid nodules; and 94.1% and 33.3%, respectively for oral cavity tumors. MS method reacts positively to solid malignant tumor and negatively to benign tumor and normal tissue (from margin samples). Highest sensitivity was found for breast and oral cavity malignancy, and high specificity was found for thyroid cancers.

**Conclusion:**

This study provided an alternative staining method for intraoperative macroscopic surgical margin evaluation, especially for rural areas without frozen section facilities.

## Introduction

Surgical margin is one of the most important prognostic factors in patients undergoing wide excision in solid malignant tumor (Chaturvedi et al., 2014; Mair et al., 2017). Local recurrence often occurs if the surgical margin is inadequately clear of malignant cells (Chaturvedi et al., 2014; Kim et al., 2014; Mair et al., 2017). The gold standard for intraoperative assessment of surgical margin is frozen section (FS). However, machines used for frozen section, such as Cryostat, are expensive, limiting their availability in many hospitals. Before microscopic evaluation, margins in solid tumor resection often depend on intraoperative gross examination (GE) performed by the surgeon. Therefore, Mair conducted a study on 435 oral cancer patients to compare the surgical margin determined with or without frozen section, and found that the sensitivity and specificity of a surgeon’s GE (without FS) were 61.9% and 88.3%, respectively (Mair et al., 2017).

Vital staining is an alternative method of staining living cells without resulting in cellular death. This method is used to distinguish normal cells and neoplastic cells, and therefore, is often used for cancer screening or as a guidance for biopsy in lesions suspected of malignancy (Bagalad and Kumar, 2013). Visual Inspection with Acetic Acid (VIA) is an established vital staining method typically performed as a screening for cervical lesions through observing acetowhitening reaction on the squamous columnar junction of the cervix (IARC, 2017). Sankaranarayanan et al., (2013) investigated diagnostic accuracy of acetic acid 4% (VIA) and Lugol’s iodine (VILI) in cervical cancer screening for 4,444 women in India. Results showed that staining methods had a relatively high sensitivity, 88.6% (VIA) and 87.2% (VILI), and specificity, 78% (VIA) and 84.7% (VILI). Sauvaget et al. (2011) conducted a meta-analysis by collecting data from 26 study of cervical cancer screening and reported an 80% sensitivity and 92% specificity for VIA.

However, vital staining is very rarely used for screening of other solid tumors. In this study, we aimed to verify whether acetic acid can be used to differentiate malignant and non-malignant lesions in solid tumor and tissue specimen in general. In addition to acetic acid, we also used iodine for second staining, which has been found to be sensitive for staining healthy tissue or non-malignant lesions. We hoped that the result of this study can be used as a benchmark to determine the use of this staining method in the evaluation of surgical margin intraoperatively.

## Materials and Methods

This is a cross-sectional diagnostic study conducted on surgical specimen taken from patients who underwent surgery or biopsy in the Surgical Oncology Division of Faculty of Medicine, Universitas Indonesia/Rumah Sakit Cipto Mangunkusumo (FMUI/RSCM). This study has been approved by the Ethical Committee of FMUI/RSCM with Protocol Number 17-11-1115. The patients were informed about the study and consent was obtained prior to the procedure. From 153 patients with solid tumors (breast, thyroid, oral, skin, maxilla/mandible, lymph node, parathyroid, soft tissue, and sarcoma) who were operated in RSCM, Jakarta, Indonesia from December 2017 to April 2018, 3 patients were excluded due to very small specimen (measuring less than 5 mm), necrotic tissue, and refusal to participate in this study, resulting in a total of 150 patients. The method used in this study was based on Visual Inspection Acetic Acid (VIA) and Visual Inspection with Lugol Iodine (VILI) method established by the World Health Organization (WHO) (IARC, 2017). The author visually inspected and compared the pre- and post-staining coloration changes in the samples compared to the unstained controls. Samples were tissue specimens taken from surgery or biopsy. From the surgical specimens, samples were obtained from the tumor and the normal tissue margin. Only tumor samples were taken from biopsy specimens. All samples were divided into 2 parts symmetrically in order to obtain the most similar specimens between the stained sample and the unstained controls. The MS samples were sprayed with 10% acetic acid, observed for one minute, and then rinsed with distilled water (aquadest). They were subsequently sprayed with iodine and then observed for one minute, and rinsed again with distilled water. Areas with acetowhitening (where the color changed to white) were classified as positive result. Areas of tissue that became yellowish-brown in vital staining is classified as negative result. Photographic documentation is made after multi-staining (MS) by putting the multi-stained and non-stained samples side by side on a clean gauze to directly compare acetowhitening reaction, yellowish-brown, or mixed changes in coloration. Subsequently, all samples were drowned in buffered formaldehyde immediately in under one hour after surgery to minimize warm ischemic process and were prepared for histopathology examination. The pathologist was blinded to group assignment and we used blinding codes in the histopathology slides.

The interpretation of histopathology result was divided into 6 groups (malignant focus, malignant, benign, normal, infection/inflammation, and undetermined). Results interpreted as malignant or malignant foci are considered positive, while other interpretations are considered negative. After all the histopathology examinations are completed, the blinding codes were removed and the results were recorded. The histopathology examination results of multi-stained samples and non-stained samples were compared to evaluate the effect of MS in pathology slide quality and necrotic effect. 

The results of acetowhitening observation were then compared with the histopathology results. The author also examined the effect of staining and slide fixation on the histopathology of multi-stained specimens compared to the non-stained group, from which we determine whether the specimen slides were in optimum or suboptimum condition. We also examined the effect of staining on cellular damage. If general necrosis was present on the treatment sample slides but not on the non-stained slides, the specimen was considered as damaged from staining; necroses present focally or around the tumor focus were considered general tumor necroses as opposed to a reaction to staining.

Since this study is quite novel study for solid tumors, the sampel size is calculated by the ability of frozen section to predict specificity of 90% compare with histopatology, with 5% of significance level and 5% precision. Data analysis was performed using with IBM SPSS version 25.0 for statistical analysis.

## Results

*Patients Characteristics*

**Figure 1:**
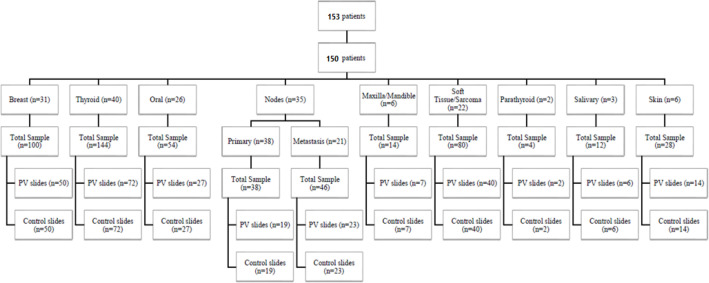
Flow of Participant

From 153 patients, 3 were excluded due to refusal to participate, specimen samples being too small and due to tissue necrosis (Figure 1). Of the remaining 150 patients, 50 were males and 100 were females. Participants’ age ranged from 10 years old to 79 years old, and the mean age was 45 years old. The most frequent case in this study was thyroid tumors, and the least frequent was parathyroid tumors. In Table 1, we present the distribution of positive values from acetic acid and iodine staining examination based on tumor type, histopathology results, and vital staining.

We took 520 biopsy samples from 150 patients and divided the samples into 2 groups: control (unstained) group and study (MS) group. The results of solid tumor samples histopathology examinations were interpreted as benign tumor, malignant focus, malignant, infection/inflammation, normal, and undetermined. Samples of healthy tissue were taken if the technique used in the surgery includes removal of healthy tissue area (e.g. in mastectomy, the sample was taken from breast cancer area and macroscopic normal breast tissue). Samples of healthy tissue were not taken for examination if only biopsy was performed. From a total of 22 metastatic lymph node specimens, 14 were metastases from breast tumors, 3 were metastases from thyroid tumors, 3 were metastases from oral cavity tumors and 2 were from skin tumors.

*Histopathology*

Table 2 showed some examples of macroscopic changes that can be seen with MS methods. Acetowhitening reactions occur in breast cancer specimens, squamous cell carcinoma, thyroid cancer, oral cancer, malignant lymphoma, and non-melanoma skin cancer. For sarcoma, the majority of staining results are mixed (acetowhitening was predominant with scattered yellowish iodine area). For this type tumor, MS generated yellowish changes in coloration in benign or normal tissue sample because iodine absorption is more dominant with no apparent acetowhitening.

The two rightmost columns showed the histopathological comparison between unstained controls and MS samples. It was apparent that the histopathological features of the unstained controls and MS samples were quite similar. No interference in terms of hematoxylin-eosin (HE) dye uptake was observed in the cell structures, including nuclei and cytoplasm, most likely due to the fact that acetic acid was also used in the process of HE staining. Therefore, MS methods will not affect the histopathological interpretation by the pathologist.

*Diagnostic value of multistaining*

Table 3 shows the comparison between positive and negative results yielded through multistaining method and histopathological examinations. Samples were insufficient to conduct statistic test for maxillary/mandibular, oral, soft tissue, parathyroid, and thyroid tumors. The sensitivity, specificity, PPV, NPV, and accuracy of multistaining method are shown for all tumors also according to tumor type. Highest sensitivity was found for breast and oral cavity tumors, and high specificity was found for thyroid tumors.

Some of the samples also have frozen section results. In surgical oncologic surgery, the using of frozen section is limited for several important occasions, like if the preoperative FNAC/biopsy is not clear, to define free margins, to define regional lymph node metastasis, etc. All the frozen sections datas, which related with the study, are collected and analyzed statistically compare with histopatological results. Table 4 shows the strenght of multistaining methods compare with frozen section methods. 

**Table 1:** Patients Characteristics

No	Variable	Category	Total patient		Total
			n	%	
1	Classification of solid tumor	Breast	31	20.6	150
		Thyroid	40	26.7	
		Oral	26	17.4	
		Skin	8	5.3	
		Primary Lymph Node	14	9.3	
		Maxilla/Mandible	6	4.0	
		Soft tissue/Sarcoma	20	13.4	
		Salivary gland	3	2.0	
		Parathyroid	2	1.3	
2	Classification of histopathology examination result	Malignant focus	23	4.4	520
		Malignant	205	39.4	
		Benign	104	20.0	
		Normal Tissue	123	23.7	
		Inflammation/infection	46	8.8	
		Undetermined	19	3.7	

**Table 2:** Comparison of Several Macroscopic and Microscopic Solid Tumor Specimens

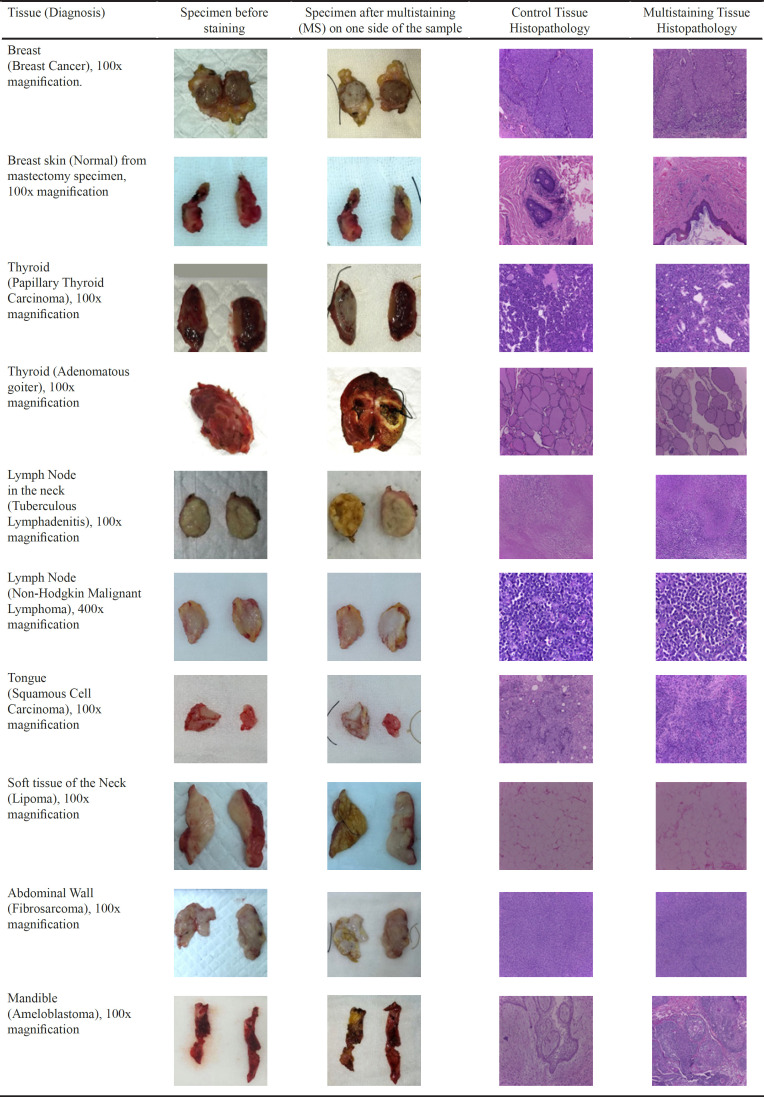

**Table 3:** Sensitivity, Specificity, PPV, NPV of MS According to Tumor Type

Category	Sensitivity (% (95% CI))	Specificity (% (95% CI))	PPV (% (95% CI))	NPV (% (95% CI))	Accuracy (% (95% CI)
All solid tumor	82 (76.6-87.4)	63.5 (56.7-70.2)	65.8 (59.1-72.4)	80.5 (74.9-86.1)	72 (65.1-78.2)
Breast	100 (75.8-100)	79.3 (61.6-90.2)	66.8 (43.8-83.7)	100 (85.7-100)	85.4 (70.8-94.4)
Thyroid	65.7 (49.2-79.2)	83.3 (55.2-95.3)	92 (75-97.8)	45 (30.8-59.2)	70.2 (55.1-82.7)
Oral	94.1 (71.3-99.9)	33.3 (0.8-90.6)	88.9 (78-94.7)	50 (7.7-97.1)	85 (62.1-96.8)
Sarcoma	80 (68.4-91.6)	73.1 (60.3-85.9)	53.3 (38.9-67.7)	90.5 (82-99)	75 (57.8-87.8)
Regional lymph node	75 (56-94)	41.7 (20-63.3)	46.2 (24.4-68)	71.4 (51.6-91.2)	55 (31.5-76.9)

PPV, Positive Predictive Value; NPV, Negative Predictive Value; 95% CI, 95% Confidence Interval

**Table 4:** Sensitivity, Specificity, PPV, NPV of MS (All Tumor) Compare with Frozen Section Datas

Category	Sensitivity (% (95% CI))	Specificity (% (95% CI))	PPV (% (95% CI))	NPV (% (95% CI))	Accuracy (% (95% CI))
Multistaining	82 (76.6-87.4)	63.5 (56.7-70.2)	65.8 (59.1-72.4)	80.5 (74.9-86.1)	72 (65.1-78.2)
Frozen section	91.7 (73-98.9)	92.9 (66.1-99.8)	95.7 (76.8-99.3)	86.7 (63.1-96.1)	92.1 (78.6 – 98.3)

PPV, Positive Predictive Value; NPV, Negative Predictive Value; 95% CI, 95% Confidence Interval

## Discussion

The current study used 10% acetic acid and iodine as staining agents, which are relatively safe for tissue and easily-available in a very affordable price. To minimize bias which might occur due to mixed/indeterminate results in the MS samples, we directly compared multi-stained samples with their non-stained counterparts placed side by side on a clean gauze. According to the 2012 College of American Pathologists (CAP) Laboratory Accreditation Manual, the MS process should be performed within 1 hour after the surgical specimens were removed from the body and the samples should be drowned in a buffer formaldehyde solution to reduce the risk of tissue necrosis related to warm ischemic time (Sharkey, 2012). Based on the analysis made by the pathologist consultant, 100% of the slides are in optimum condition for assessment and no necrosis related to MS effect was found in the multi-stained samples when compared to the non-stained samples.

Based on the result of this study, we can conclude that this method of staining (using acetic acid and iodine) can be used to differentiate between malignant solid tumor tissue and normal tissue in general, with a sensitivity and specificity of 82% and 63.5%, respectively. If mixed result were included in the positive group, the sensitivity was increased to 86% (95% CI 81.7 – 90.3), and specificity was decreased to 47.8% (95% CI 41.8 – 54.2) for all solid tumors (breast, thyroid, oral, skin, salivary gland, soft tissue, parathyroid, lymph node, maxillary and mandible tumors).

When broken down into subgroups, the highest sensitivity was found in breast and oral cavity tumors, and the highest specificity was found in thyroid tumors. In patients with solid tumor of the breast, MS method was found to have a sensitivity and specificity of 100% (95% CI 75.8 – 100) and 79.3% (95% CI 61.6 – 90.2), respectively. The color of all samples taken from normal breast tissue from surgical specimen turned to yellow, indicating negative MS results. This result can be used in the future to analyze the role of MS method in the surgical margin examination during breast cancer surgery.

In patients with oral cavity tumors, we found a sensitivity, specificity, PPV, and NPV of 94.1%, 33.3%, 88.9% and 50%, respectively. Watanabe et al. evaluated the effectiveness of iodine staining in detecting oral cancer (squamous cell carcinoma) and found a sensitivity and specificity of 100% and 59,6%, respectively (Watanabe et al., 2012). In oral cavity carcinoma, most of the samples were obtained from biopsy due to the fact that patients typically came at an advanced stage, and had been given neoadjuvant therapy in the form of chemotherapy or chemoradiation. Surgery would then be performed if the patient responded well to neoadjuvant, either through partial response or complete response. Of all patients with oral cavity carcinoma enrolled in this study, only two underwent definitive surgery; thus, only two normal tissue sample (surgical margin) were obtained. This MS method can be used in further study for oral cancer screening. 

In patients with solid tumor of the thyroid, we found a sensitivity and specificity of 65.7% (95% CI 49.2 – 79.2%) and 83.3% (95% CI 55.2 – 95.3%), respectively. If mixed results were included in the calculation, the values became 73.9% (95% CI 63.4 - 84.6) and 50% (95% CI 29.9 – 70.1%) for sensitivity dan specificity, respectively. The fact that specificity surpassed sensitivity meant vital staining had sufficient ability to differentiate normal thyroid and malignant thyroid. If the thyroid specimen (from isthmolobectomy and lobectomy surgery) did not exhibit acetowhitening reaction with vital stain, it could strengthen the clinical intraoperative judgment that the particular case is a non-malignancy case. This can be used as a consideration to not performing total thyroidectomy if the contralateral lobe does not appear abnormal.

In cases with primary lymph node metastasis, we found a sensitivity, specificity, PPV, and NPV of 80%, 42.9%, 50%, and 75%, respectively. Calculation of confidence interval was not performed due to the samples being less than 20, as a result of the rarity of lymphoma cases. It is interesting to observe that lymphoma also gave positive acetowhitening result. Meanwhile, in patients with regional lymph node involvement, we found a sensitivity and specificity of 71.4% and 45.5%, respectively. If mixed results were included in the analysis, the total number of samples reached 20, and the confidence interval can be calculated. The sensitivity and specificity then became 75% and 41.7%, respectively. Our analysis was performed on the specimens taken from patient with enlarged regional lymph nodes instead of all patients. Our consideration was that the enlarged lymph nodes were important for histopathologic analysis and for further therapeutic decision for the patients. Based on this result, we can conclude that MS is sensitive enough to detect metastasis to regional lymph node, but not specific enough to rule out metastasis of regional lymph node. Since this MS method has been proven in this study to not interfere with histopathological interpretation, further study can be done to evaluate its use for the detection of regional lymph node metastasis.

In patients with sarcoma, we found a sensitivity and specificity of 60% and 79.8%, respectively. If mixed results were included in the calculation, the values became 80% and 73.1%, respectively. Mixed results predominantly occurred in the sarcoma specimens in the first minute after MS application. However, after 3-5 minutes, the yellowish-brown color faded and acetowhitening became dominant. As a malignant mesenchymal tumor, sarcomas have been known to have an intratumor dendritic cell arranged like a lace. This mature dendritic cell contains fat and glycogen (Subbiah et al., 2018). In relation to vital staining, mixed-stain is likely to be dominant in sarcomas due to iodine binding to glycogen in dendritic cells. However, acetowhitening reaction still occurred in cells obtained from patients with sarcoma (Table 2).

We found in this study that almost all types of cancer cells react to acetic acid and do not react to iodine, underlying the different general biochemical conditions between solid malignant tumor cells and normal tissue. Cancer cells may have different biochemical concentration compared to normal cells or they may not have some of the molecular components which are present in normal cells (Mady and Al-Shihry, 2018). 

This study also compare the MS methods to FS, since both of them have intraoperative (fast) results. The sensitivity of FS is 91,7% higher than MS (82%), NPV of FS is 86,7% comparable with MS (80,5%). This is shown that MS methods can be promising to define surgical free margin if the FS is not available. Beside that the MS results are 5-10 times faster than FS results intraoperatively. But this results need further study, spesifically for certain type of malignant tumors. 

Warburg, a pioneer of cancer biochemistry, stated that: 1) all cancers experience an increase in the speed of glycolysis; and 2) glycolysis in aerobic conditions (high aerobic glycolysis) occurs due to defects in the cell’s respiration system. In observing the Warburg effect, most of cancer cells produce energy with an increase in glycolysis followed by lactic acid fermentation in the cytosol. While in normal cells glycolysis velocity is low, energy is more important than pyruvate oxidation in mitochondria (Alfarouk et al., 2011; Alfarouk et al., 2014). Since iodine binds to intracellular glycogen (Kaczor, 2014), and the majority of cancer cells are glycogen depleted, it stands to reason that iodine cannot exhibit staining effect (IARC, 2017). 

Acetic acid has been used for centuries to detect precancerous cervical lesions through acetowhitening reactions, specifically the discoloration of neoplastic lesions to white. Acetic acid is also useful for detecting oral cavity cancer. Some hypotheses related to the occurrence of acetowhitening include the precipitation of core proteins supported by the fact that metaplastic tissue has increased core protein. In addition, cytokeratin expression is hypothesized to have an essential role in the occurrence of acetowhitening. A study conducted by Marina, et al. on the effect of acetic acid on light emission from cells found that precipitation of core cell protein and cytoplasm acted as a cause of acetowhitening in cancer cells, but the effect was stronger on the nucleus. Protein in nucleus of cancer cells being higher than normal cells is related to high rate of mitosis in cancer cells. Thus, the effect of protein precipitation by acetic acid is more pronounced on cancer cells (Marina, 2012).

Based on the results of the current study, we try to predict malignant cell structure that reacted with acetic acid. Some of the cell structure commonly used for immunohistochemistry include, among others, cytokeratin, vimentin, desmin, laminin, neurofilament, and s100. However, the suspected mutated gene is KRAS, due to the fact that its generally appears (85%) in malignant solid tumor (Calaf and Abarca-Quinones, 2016). Biomolecular research is needed to prove this hypothesis.

This is a baseline study to screen general solid tumor reaction to MS method using 10% acetic acid and iodine. Surprisingly, many solid tumors gave positive results and normal tissue samples from surgical margin/specimen gave negative results. Limitations of this study are some solid tumor are rare, like parathyroid tumor, salivary gland tumor, maxilla/mandible bone tumor, and skin cancer. Other limitation is lack of previous researches for comparison to this study since this method are generally used for cervical cancer screening or oral cancer screening. Further studies for the role of MS in screening or in gross examination of surgical margin can be done specifically based on organ/tumor type. We hoped that the results of this study can be used in clinical practice, especially for hospitals in rural areas with no frozen section facilities.

MS provided clear macroscopic (visual) differentiation between malignant and non-malignant tissue but did not interfere with HE staining for microscopic examinations, most likely due to the fact that acetic acid was used in the process of HE staining. Biomolecular study is needed to identify the specific protein of malignant cancer cell which reacts to acetic acid. Specific diagnostic studies focusing on specific types of solid tumor are needed, as well as studies on the potential of MS for the determination of macroscopic surgical margin of malignant solid tumor.
